# Regular Alpha-Fetoprotein Tests Boost Curative Treatment and Survival for Hepatocellular Carcinoma Patients in an Endemic Area

**DOI:** 10.3390/cancers16010150

**Published:** 2023-12-28

**Authors:** Joo Hyun Oh, Jonghyun Lee, Eileen L. Yoon, Soung Won Jeong, Soon Sun Kim, Young Eun Chon, Sang Bong Ahn, Dae Won Jun

**Affiliations:** 1 Department of Internal Medicine, Nowon Eulji Medical Center, College of Medicine, Eulji University, Seoul 01830, Republic of Korea; 20210372@eulji.ac.kr; 2 Department of Internal Medicine, College of Medicine, Hanyang University, Seoul 04736, Republic of Korea; jonghyunlee1993@gmail.com (J.L.); mseileen80@gmail.com (E.L.Y.); 3 Department of Internal Medicine, Soonchunhyang University Seoul Hospital, College of Medicine, Soonchunhyang University, Seoul 04401, Republic of Korea; jeongsw@schmc.ac.kr; 4 Department of Gastroenterology, School of Medicine, Ajou University, Suwon 16499, Republic of Korea; cocorico99@gmail.com; 5 Department of Gastroenterology, CHA Bundang Medical Center, CHA University, Seongnam 13496, Republic of Korea; nachivysoo@chamc.co.kr

**Keywords:** alpha-fetoprotein, hepatocellular carcinoma, chronic hepatitis B

## Abstract

**Simple Summary:**

This study evaluated the impact of alpha-fetoprotein (AFP) testing frequency on survival rates in hepatocellular carcinoma (HCC) patients. Analyzing data from 81,520 patients in Korea, it was found that increased AFP testing significantly improved survival, particularly in patients with hepatitis B undergoing antiviral treatment. Those tested three or more times before diagnosis had a higher likelihood of receiving curative treatments, such as liver transplantation. The study suggests that combining AFP tests with ultrasound screenings could better detect HCC early, offering enhanced treatment opportunities and improved survival chances, especially for hepatitis B patients.

**Abstract:**

Guidelines vary on alpha-fetoprotein (AFP) testing for hepatocellular carcinoma (HCC) screening. This study aims to reassess AFP’s role in HCC surveillance, utilizing a comprehensive, recent, nationwide cohort. Utilizing the National Health Claims Database from the Korean National Health Insurance Service, this research included data from 185,316 HCC patients registered between 2008 and 2018. Specifically, 81,520 patients diagnosed with HCC from 2008 to 2014 were analyzed. The study focused primarily on mortality and, secondarily, on the status of curative treatments. Multivariate analysis revealed that frequent AFP testing significantly impacts overall survival in HCC patients. Specifically, each additional AFP test correlated with a 6% relative improvement in survival (hazard ratio = 0.94, 95% CI: 0.940–0.947, *p* < 0.001). Patients who underwent AFP testing three or more times within two years prior to HCC diagnosis showed improved survival rates, with 55.6% receiving liver transplantation or hepatectomy. This trend was particularly pronounced in hepatitis B patients undergoing antiviral treatment. The findings highlight the potential of regular AFP testing to enhance survival in HCC patients, especially those with hepatitis B. Integrating frequent AFP testing with ultrasonography could increase the likelihood of early detection and access to curative treatments.

## 1. Introduction

Hepatocellular carcinoma (HCC) is the most common type of primary liver cancer, with the fifth highest prevalence rate in men and the seventh highest in women [1,2]. Liver cancer also ranks as the second most common cause of premature cancer-related deaths [3]. According to the GLOBOCAN 2020 report, approximately 905,700 people were diagnosed with liver cancer, and 830,200 people died from the disease globally [4]. One of the primary reasons for the high mortality rates associated with HCC is delayed diagnosis, often occurring at advanced stages of the disease. Early detection plays a crucial role in improving the chances of receiving curative treatment and enhancing survival rates for individuals with HCC [5]. Increasing the emphasis on early detection and encouraging individuals at high risk, such as those with chronic liver diseases or a history of liver cancer, to undergo regular screening can contribute to improving HCC outcomes [6]. Timely diagnosis allows for the implementation of appropriate treatment strategies and potentially extends the survival time of affected individuals.

Alpha-fetoprotein (AFP) is commonly used as a biomarker for HCC screening. However, its performance in accurately diagnosing HCC remains uncertain [7]. AFP testing is not considered a standalone screening tool for HCC surveillance in the United States and Europe due to its insufficient diagnostic accuracy for detecting HCC derived from very limited data [8,9]. Several studies have reported on whether additional AFP tests were effective for the early detection of HCC. However, most of these studies suffer from small sample sizes and issues with study design [10,11,12]. Hence, there is a pressing need for a large-scale study with longitudinal data to ascertain the efficacy of AFP testing in the early detection of HCC.

Additionally, no significant reports have conclusively demonstrated that AFP testing contributes to improved survival rates in HCC patients. A randomized controlled trial showed the feasibility of using AFP for early HCC diagnosis, but it did not demonstrate a significant reduction in mortality rates [13]. This discrepancy might be attributed to the limitation in selecting suitable treatment methods, even when an early diagnosis of HCC is established. However, it is worth noting that the landscape of HCC treatment has been evolving with loco-regional therapy [14] and systemic therapies [15]. These advancements have resulted in improved survival rates after a diagnosis of HCC [16] and may hold promise for refining the clinical utility of AFP testing. Advancements in antiviral therapy [17] and the management of underlying liver diseases can also affect the reliability of AFP testing as a screening tool. Therefore, considering the advancements in the field, it appears to be the proper time to conduct a comprehensive reappraisal of the role of AFP testing in HCC surveillance, particularly in determining its effectiveness in improving survival rates and facilitating curative treatments for HCC patients.

Thus, the objective of this study was to comprehensively assess the possible role of regular AFP testing in patients at high risk for HCC using a nationwide database.

## 2. Materials and Methods

### 2.1. Study Design

This was a retrospective cohort study. We extracted data from the KNHIS from January 2005 to December 2018. Patients who were newly diagnosed with HCC with International Classification of Diseases, tenth revision (ICD-10), codes C220 and V193 were identified in the KNHIS database and newly diagnosed with HCC during the period from January 2008 to December 2014. This study protocol was approved by the Institutional Review Board of Hanyang University, which granted a waiver for the requirement for informed consent due to the exclusive use of de-identified data (IRB file number 2019-07-014).

### 2.2. Data Source: National Health Insurance Service

The KNHIS is the official and comprehensive national healthcare insurance program of Korea [18]. It operates as a single-payer system, providing coverage for almost the entire population of the country (97.2%). One of its primary functions is the maintenance of national records, which include details on all insurance-covered inpatient and outpatient visits, procedures, and prescriptions. KNHIS claims for patient visits, procedures, and prescriptions are coded using Korean Drug and Anatomical Therapeutic Chemical Codes (KDATCC) and the ICD-10. Information such as lab data and stages of the disease were not available in the dataset.

### 2.3. Study Population

Patients were excluded if they met any of the following criteria: (1) had a diagnosis of HCC between 2005 and 2006 due to concerns regarding the reliability of data during that period, (2) had a diagnosis of HCC within the 2-year period prior to the index date, (3) had a diagnosis of malignancies other than HCC, as identified by ICD-10 codes other than C220 and V193, (4) had a history of organ transplantation, (5) had human immunodeficiency virus infection, or (6) had missing patient identification information, including age or sex.

### 2.4. Clinical Variables

The study investigated the baseline characteristics of patients who were registered with newly diagnosed HCC. These characteristics included age, gender, etiologies, the presence of cirrhosis, diabetes mellitus (DM), hypertension, dyslipidemia, and other comorbidities, as well as the initial treatment for HCC. The study also examined the frequency of medical visits and hospitalizations, abdominal computed tomography (CT) scans within 2 years of the HCC diagnosis, the use of antiviral therapy, and the frequency of blood tests, such as AFP. However, medical visits and blood tests within 30 days of the HCC diagnosis were excluded as they were considered to be for the purpose of the HCC diagnosis.

### 2.5. Operational Definitions

We obtained information on chronic hepatitis and its causes from the database of medical treatments. Based on the HCC cause, patients were classified into hepatitis B virus (HBV)-related, hepatitis C virus (HCV)-related, alcohol-related, and others-related HCC. The use of antiviral therapy during the follow-up period was identified using KDATCC codes, including lamivudine, adefovir, entecavir, tenofovir disoproxil fumarate, tenofovir alafenamide, tenofovir disoproxil aspartate, telbivudine, clevudine, and besifovir for HBV, and ribavirin and interferon for HCV. The presence of comorbidities was determined by ICD-10 codes and then quantified using the Charlson Comorbidity Index (CCI). The initial treatment for HCC included liver transplantation, hepatectomy, local ablation therapy, transarterial chemotherapy, sorafenib, radiotherapy, best supportive care, and others. A more detailed description of each definition, including the relevant ICD codes and KDATCC codes, is available in Supplementary Table S1.

### 2.6. Definition of Outcomes

The primary outcome of this study was mortality. The secondary outcome was treatment status, specifically curative treatment. Curative treatment encompassed liver transplantation, hepatectomy, and local ablation therapy when used as an initial treatment. Patient follow-up was conducted until 31 December 2018, or the date of death, in accordance with the study’s timeline.

### 2.7. Statistical Analysis

Data are reported as the median value with the interquartile range or as frequencies with corresponding percentages. Patients were stratified based on etiologies, including HBV, HCV, alcohol-related, and other causes. Analysis of variance was employed to assess differences among groups of continuous variables, while chi-square testing was used for categorical variables. Survival rates, categorized by the number of AFP tests, were compared using Kaplan–Meier curves. The proportion of patients receiving curative treatment was visually depicted according to the number of AFP tests. Cox proportional hazards model analysis was conducted to explore the impact of each variable on survival. The model was adjusted for gender, age, the presence of cirrhosis, DM, hypertension, dyslipidemia, CCI, initial HCC treatment (curative or non-curative treatment), number of AFP tests, and antiviral treatment. Analysis of antiviral treatment was conducted only in patients with either HBV or HCV.

A sensitivity analysis was performed among patients with HBV or HCV, considering the presence or absence of antiviral treatment, to examine the effect of the AFP testing frequency on survival. All statistical analyses were performed using R software (version 4.0.2). Two-sided *p*-values < 0.05 were considered statistically significant.

## 3. Results

### 3.1. Study Population

**Figure 1:**
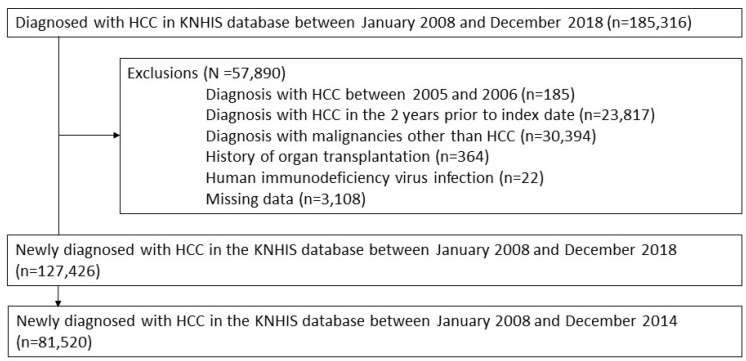
Participants.

We screened a total of 185,316 adult men or women who participated in the KNHIS from January 2008 to December 2018. Among them, we excluded 57,890 participants who met the following exclusion criteria: (1) HCC diagnosis between 2005 and 2006 (*n* = 185), (2) HCC diagnosis in the 2 years prior to the index date (*n* = 23,817), (3) diagnosed with malignancies other than HCC (*n* = 30,394), (4) history of organ transplantation (*n* = 364), (5) human immunodeficiency virus (HIV) infection (*n* = 22), and (6) missing data (*n* = 3108). Consequently, 127,426 patients were newly diagnosed with HCC between January 2008 and December 2018, without HCC within 2 years before the index date, other cancers, organ transplantation, or HIV infections. Among them, a total of 81,520 participants diagnosed with HCC between January 2008 and December 2014 were analyzed (Figure 1).

### 3.2. Baseline Characteristics

The baseline characteristics of all patients are summarized in Table 1. In total, 81,520 patients who were newly diagnosed with HCC were included. The highest proportion of HCC cases was attributed to HBV (65.8%), followed by other causes (14.9%), HCV (10.3%), and alcohol consumption (9.0%). The patients were predominantly male, and the age at diagnosis was relatively lower in the HBV group. As Table 1 illustrates, transarterial chemotherapy was the most commonly utilized initial treatment post-HCC diagnosis, followed by best supportive care. In the HBV cohort, AFP tests were performed an average of 2.42 times, and CT scans were performed an average of 2.11 times, indicating the highest frequency among the etiology cohorts (Table 2).

### 3.3. Number of AFP Tests Was an Independent Risk Factor for Overall Survival in Patients with HCC

The multivariate analysis showed that gender, age, the presence of cirrhosis, DM, hypertension, dyslipidemia, CCI, receipt of curative HCC treatment, and the frequency of AFP testing were independent risk factors for overall survival in patients with HCC (Table 3). Notably, an increase in the number of AFP tests conducted was associated with a 6% relative improvement in survival (hazard ratio (HR) = 0.94, 95% confidence interval (CI): 0.940–0.947, *p <* 0.001). When conducting a subgroup analysis of the viral hepatitis cohort, the number of AFP tests performed (HR = 0.96, 95% CI: 0.95–0.96), as well as receiving antiviral treatment, were identified as independent protective factors for developing HCC (Table 3).

### 3.4. Nearly Half of the Patients Who Had Undergone AFP Testing Underwent Curative Treatment

**Figure 2:**
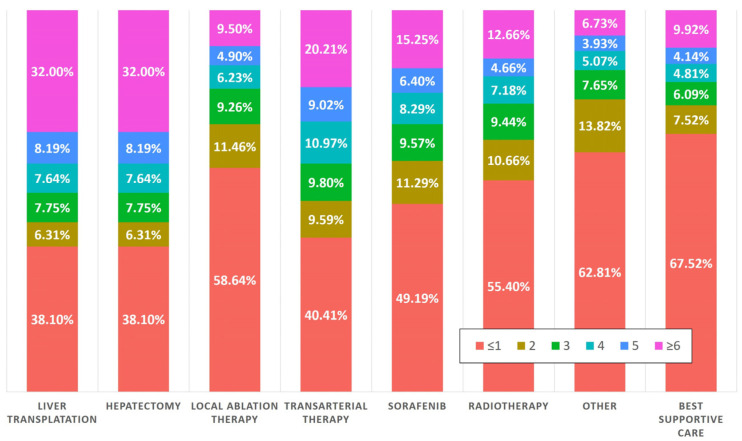
Initial treatment patterns in HCC patients based on prior AFP testing frequency.

During the two-year period prior to their diagnosis with HCC, 55.6% of patients who underwent three or more AFP tests received liver transplantation or hepatectomy as their initial treatment modality. In the subset of patients who had four or more AFP tests, 47.8% were treated with curative intent. Additionally, 38.1% of patients who underwent curative treatment had one or fewer AFP tests during the two years before diagnosis. Conversely, there was an observable trend toward non-curative treatments among patients with less frequent AFP testing. Specifically, 49.19% of patients who were initially treated with sorafenib had undergone one or fewer AFP tests in the preceding two years. Similarly, 67.5% of patients who received best supportive care as their initial treatment approach had the same minimal testing frequency, as illustrated in Figure 2.

### 3.5. Three or More AFP Tests in Two Years Were Associated with Decreased Overall Survival

**Figure 3:**
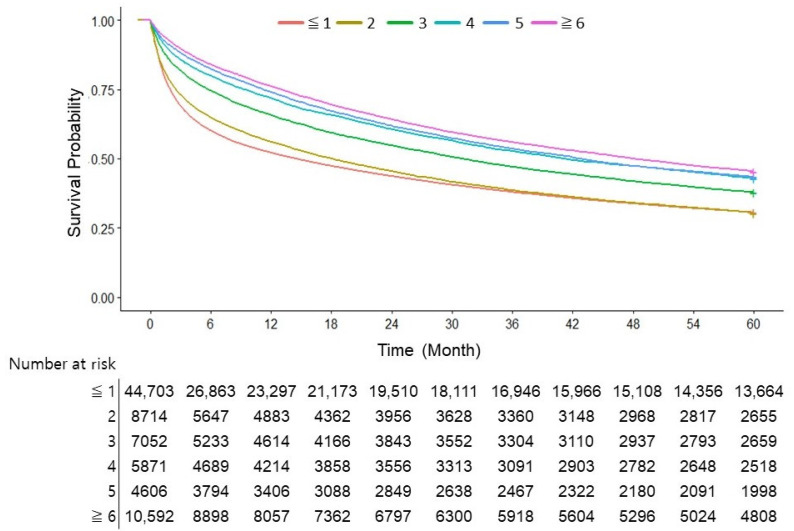
Survival curve according to the number of AFP tests in the overall cohort.

**Figure 4:**
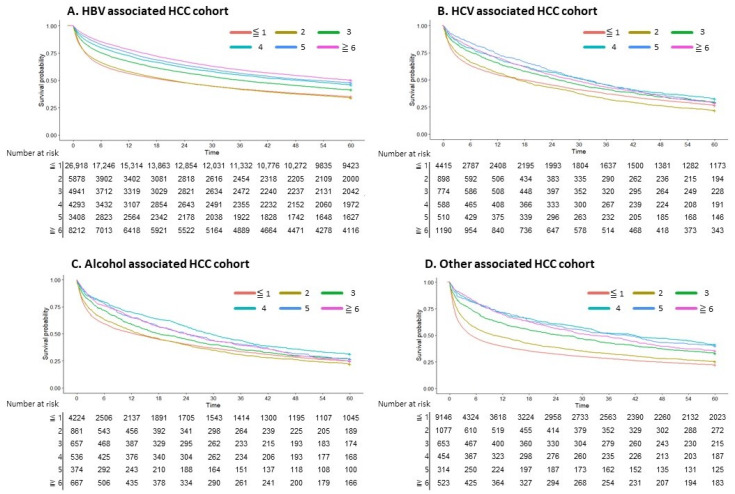
Survival curve according to the number of AFP tests in underlying diseases. (**A**) HBV-associated HCC cohort. (**B**) HCV-associated HCC cohort. (**C**) Alcohol-associated HCC cohort. (**D**) Other-associated HCC cohort.

A notable increase in survival starting from the third AFP test was seen in the overall cohort, with a gradual improvement observed as the number of tests increased (*p <* 0.001; Figure 3). When examining the data based on the underlying causes of HCC, patients with HBV demonstrated a distinct survival improvement as the number of AFP tests increased. No significant difference was observed between undergoing one or two tests (*p =* 0.51). However, survival increased progressively in patients who underwent three or more tests. In cases of HCV- and alcohol-associated HCC, a significant survival benefit was confirmed in patients who underwent AFP testing three or more times. For other HCC cases, a significant survival benefit was confirmed with two or more AFP tests (Figure 4).

### 3.6. AFP Testing in Viral Hepatitis-Associated HCC

**Figure 5:**
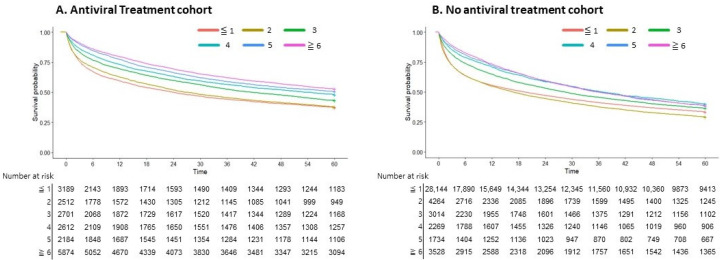
Survival curve according to the number of AFP tests in patients with and without receiving antiviral treatment. (**A**) Antiviral treatment cohort. (**B**) No antiviral treatment cohort.

When the data were stratified in the HBV and HCV cohorts based on antiviral treatment, a proportional improvement in survival was seen as the number of AFP tests increased among patients who received antiviral therapy (*p <* 0.001; Figure 5). Measuring AFP three or more times within the two years prior to being diagnosed with HCC in viral hepatitis cases was associated with decreased overall survival.

## 4. Discussion

This study showed the clinical importance of AFP testing in HCC screening in an endemic area. Frequent AFP testing was independently linked to enhanced patient survival. The findings revealed an association between the frequency of AFP testing, the likelihood of receiving curative treatment at the initial diagnosis, and overall survival. Among the various etiologies of HCC patients, the most significant association between AFP testing frequency and survival was observed in HBV patients. Specifically, a clear correlation with improved survival rates was evident when AFP testing was conducted at least three times over a two-year period.

The American Association for the Study of Liver Disease [8] and the European Association for the Study of the Liver [9] guidelines recommend ultrasound as the primary surveillance method for HCC in at-risk populations. Ultrasound has limitations as an operator-dependent method, and its sensitivity in diagnosing early-stage HCC is only around 63% [19]. Therefore, combining AFP with ultrasound is commonly used to improve the sensitivity of an HCC diagnosis [20]. Previous studies showed that AFP testing alone did not show a survival benefit or sufficient sensitivity for early HCC detection. Therefore, it is not currently recommended as a standalone method of surveillance. One of the reasons for the lower sensitivity of AFP may be its elevation in chronic liver diseases [21]. However, the present study found that frequent AFP testing led to an increase in curative treatment as well as survival gain. This effect was particularly prominent in patients with HBV receiving antiviral therapy. The previously ambiguous improvement in survival due to AFP testing appears to be attributed to the enhanced effectiveness of antiviral therapy, which, when properly controlling underlying hepatitis, amplifies the diagnostic performance of AFP [22]. These findings emphasize the importance of AFP as a predictive factor in HCC prediction models that specifically target antiviral-treated HBV patients [23].

Not only has the diagnostic performance of AFP improved, but it also seems that regular and serial AFP testing have contributed to enhanced survival. Recent studies reported that serial AFP testing assisted in the early detection of HCC and increased the likelihood of individuals receiving curative treatment [23,24,25]. In our study, patients who underwent AFP tests frequently were significantly more likely to receive curative treatments, such as liver transplantation, hepatic resection, or radiofrequency ablation. Therefore, the present study supports the implementation of regular and frequent AFP testing as a beneficial strategy. Integrating routine and frequent AFP testing into the surveillance strategy ensures that HCC surveillance is extended to all patients, regardless of their ability to undergo regular ultrasound examinations.

The utility of AFP was highest in patients with HBV receiving antiviral treatment, followed by patients with HCV. However, no clear utility was observed in patients with alcohol use disorder. This is consistent with the findings of a previous nationwide cohort study in Denmark, which showed low utility of HCC surveillance in patients with alcoholic cirrhosis [26]. The disparate utility of AFP testing in viral hepatitis patients versus patients with alcohol use disorder may be attributable to differences in the etiology and pathogenesis of HCC [27,28]. For instance, HBV DNA integration into the host genome can elicit insertional mutagenesis, leading to oncogene activation and promoting carcinogenesis [29]. On the other hand, the association between alcohol consumption and HCC development is more complex, with additional factors such as concomitant liver damage and cirrhosis also playing a role [30]. According to preclinical studies, AFP expression in adult cells is known to be suppressed at the promoter and two enhancers through the action of corepressors and methylated histones [31]. In HCC, hypermethylation has been identified as a potential mechanism in HCC contributing to AFP overexpression [32]. These mechanisms may be further modulated by viral genetics. Given these findings, it is important to consider the underlying etiology of liver disease when determining the utility of AFP testing in HCC surveillance strategies. Further research is warranted to explore alternative surveillance strategies and biomarkers that may be more appropriate for the patients with alcohol use disorder.

The study had some limitations. The major limitation was that abdominal ultrasound examinations were not included in the analysis. During the data collection period of this study, abdominal ultrasound examinations were not covered by insurance, so the data were not available. Secondly, although the frequency of AFP testing was known, the actual test results were not available, which prevented us from examining the prognostic significance of AFP levels and changes. While our dataset allowed for an analysis of comorbidity burden via the CCI, it lacked the granularity to account for detailed comorbidity profiles and concurrent medication use. Additionally, our dataset did not encompass certain aspects of treatment for underlying liver diseases, such as changes in alcohol consumption, or on the protective effects of specific treatments, such as bariatric surgery [33], for other HCC cohorts. Since the study lacked validation from other groups, further validation in different populations is warranted. Lastly, a cost-effectiveness evaluation was not conducted. However, considering that AFP testing is relatively inexpensive and easy to perform, it is likely to have value as a surveillance tool, even without a separate evaluation. While the study demonstrated the potential benefits of regular and frequent AFP testing, further research is needed to validate its effectiveness and determine the optimal frequency and thresholds for AFP monitoring.

## 5. Conclusions

The present study elucidated the role of regular and frequent AFP testing in enhancing the survival rates and prospects of curative treatments for HCC patients, particularly in those undergoing antiviral therapy for HBV. While ultrasound remains a primary surveillance tool, the integration of consistent AFP testing can significantly amplify its diagnostic efficacy, offering a marked advantage. Further research is needed to fine-tune the optimal protocols for AFP monitoring, considering its potential cost-effectiveness and accessibility as a surveillance tool.

## Figures and Tables

**Table 1:** Baseline characteristics of the study participants who were diagnosed with HCC from 2008 to 2014.

		HBV *n* = 53,656	HCV *n* = 8376	Alcohol *n* = 7319	Other *n* = 12,169	*p*-Value
Male, *n* (%)		42,744 (79.66)	5830 (69.60)	6837 (93.41)	8522 (70.03)	<0.001
Age, *n* (%)						<0.001
	10~19	13 (0.02)			22 (0.18)	
	20~29	152 (0.28)	6 (0.07)	4 (0.05)	86 (0.71)	
	30~39	1680 (3.13)	35 (0.42)	72 (0.98)	163 (1.34)	
	40~49	10,042 (18.72)	366 (4.37)	480 (6.56)	647 (5.32)	
	50~59	21,274 (39.65)	1346 (16.07)	1611 (22.01)	1677 (13.78)	
	60~69	13,680 (25.50)	2487 (29.69)	2552 (34.87)	3055 (25.10)	
	70+	6815 (12.70)	4136 (49.38)	2600 (35.52)	6519 (53.57)	
Index date year, *n* (%)						<0.001
	2008	7885 (14.70)	1202 (14.35)	1052 (14.37)	2131 (17.51)	
	2009	7715 (14.38)	1197 (14.29)	951 (12.99)	1730 (14.22)	
	2010	7557 (14.08)	1166 (13.92)	989 (13.51)	1773 (14.57)	
	2011	7909 (14.74)	1238 (14.78)	1047 (14.31)	1586 (13.03)	
	2012	7532 (14.04)	1248 (14.90)	1113 (15.21)	1624 (13.35)	
	2013	7480 (13.94)	1169 (13.96)	1106 (15.11)	1661 (13.65)	
	2014	7578 (14.12)	1156 (13.80)	1061 (14.5)	1664 (13.67)	
Cirrhosis, *n* (%)		44,767 (83.43)	6858 (81.88)	6571 (89.78)	5753 (47.28)	<0.001
Diabetes mellitus, *n* (%)		24,890 (46.39)	5040 (60.17)	4733 (64.67)	6444 (52.95)	<0.001
Hypertension, *n* (%)		25,935 (48.34)	5752 (68.67)	4645 (63.46)	7596 (62.42)	<0.001
Dyslipidemia, *n* (%)		20,697 (38.57)	3639 (43.45)	3209 (43.84)	4725 (38.83)	<0.001
CCI, *n* (%)		6.76 (2.41)	7.42 (2.47)	7.54 (2.42)	7.02 (2.79)	<0.001
	low (≤6)	29,029 (54.10)	3402 (40.62)	2764 (37.76)	5596 (45.99)	<0.001
	high (>6)	24,627 (45.90)	4974 (59.38)	4555 (62.24)	6573 (54.01)	
HCC treatment, *n* (%)						<0.001
	Liver transplantation	782 (1.46)	45 (0.54)	51 (0.70)	25 (0.21)	
	Hepatectomy	7474 (13.93)	580 (6.92)	477 (6.54)	1153 (9.47)	
	Local ablation therapy ^a^	5297 (9.87)	1060 (12.66)	660 (9.02)	523 (4.30)	
	Transarterial therapy ^b^	24,649 (45.94)	3861 (46.10)	3114 (42.55)	3207 (26.35)	
	Sorafenib	2022 (3.77)	216 (2.58)	206 (2.81)	379 (3.11)	
	Radiotherapy	1545 (2.88)	190 (2.27)	144 (1.97)	419 (3.44)	
	Other ^c^	733 (1.37)	98 (1.17)	108 (1.48)	391 (3.21)	
	Best supportive care	11,154 (20.79)	2326 (27.77)	2559 (34.96)	6072 (49.90)	

Abbreviations: HBV, hepatitis B virus; HCV, hepatitis C virus; CCI, Charlson Comorbidity Index; HCC, hepatocellular carcinoma. ^a^ Local ablation therapy includes radiofrequency ablation, ethanol injection, and cryotherapy. ^b^ Transarterial therapy includes transarterial chemoembolization and transarterial radioembolization. ^c^ Other therapy includes hepatic artery infusion chemotherapy and other cytotoxic chemotherapy.

**Table 2:** Healthcare utilization of the study population within two years before HCC diagnosis.

			HBV *n* = 53,656	HCV *n* = 8376	Alcohol *n* = 7319	Other *n* = 12,169	*p*-Value
Healthcare utilization							
	Hospital admission, mean (SD)		2.83 (2.89)	3.41 (3.84)	3.66 (3.87)	3.0 (3.69)	<0.001
	Clinic visits, mean (SD)		35.90 (37.65)	59.22 (53.67)	46.13 (44.81)	49.40 (50.98)	<0.001
Blood tests, number							
	ALT, *n* (%)		5.91 (5.25)	7.06 (5.90)	6.36 (6.08)	3.85 (4.42)	<0.001
		0	7776 (14.49)	809 (9.66)	851 (24.11)	2839 (23.33)	<0.001
		1	4481 (8.35)	639 (7.63)	646 (18.30)	1773 (14.57)	
		2	4296 (8.01)	619 (7.39)	635 (17.99)	1464 (12.03)	
		3	4209 (7.87)	597 (7.13)	646 (18.30)	1206 (9.91)	
		≥4	32,894 (61.31)	5712 (68.19)	752 (21.30)	4887 (40.16)	
	AFP, *n* (%)		2.42 (2.81)	2.32 (2.82)	1.89 (2.41)	1.09 (1.91)	<0.001
		≤1	26,920 (50.17)	4416 (52.72)	4224 (57.71)	9147 (75.17)	<0.001
		2	5881 (10.96)	898 (10.72)	861 (11.76)	1078 (8.86)	
		3	4941 (9.21)	774 (9.24)	657 (8.98)	653 (5.37)	
		4	4293 (8.00)	588 (7.02)	536 (7.32)	454 (3.73)	
		5	3408 (6.35)	510 (6.09)	374 (5.11)	314 (2.58)	
		≥6	8213 (15.31)	1190 (14.21)	667 (9.11)	523 (4.30)	
	HBV DNA, *n* (%)		1.09 (1.81)	0.03 (0.19)	0.03 (0.25)	0.06 (0.33)	<0.001
		0	30,590 (57.01)	8190 (97.78)	7119 (97.27)	11,608 (95.39)	<0.001
		1	10,091 (18.81)	156 (1.86)	167 (2.28)	468 (3.85)	
		2	4629 (8.63)	24 (0.29)	24 (0.33)	53 (0.44)	
		3	2730 (5.09)	4 (0.05)	4 (0.05)	20 (0.16)	
		≥4	5616 (10.47)	2 (0.02)	5 (0.07)	20 (0.16)	
Radiologic studies, number							
	abdominal CT, *n* (%)		2.11 (2.20)	2.01 (2.06)	1.89 (2.02)	1.24 (1.71)	<0.001
		0	22,644 (42.20)	3600 (42.98)	3271 (44.69)	7054 (57.97)	<0.001
		1	7741 (14.43)	1389 (16.58)	1186 (16.20)	1954 (16.06)	
		≥2	23,271 (43.37)	3387 (40.44)	2862 (39.10)	3161 (25.98)	
Antiviral treatment							
	Yes		17,992 (33.53)	1082 (12.92)	NA	NA	<0.001

Abbreviations: HBV, hepatitis B virus; HCV, hepatitis C virus; SD, standard deviation; ALT, alanine aminotransferase; AFP, alpha-fetoprotein; HBV DNA, HBV deoxyribonucleic acid; CT, computed tomography.

**Table 3:** Univariate and multivariate Cox regression analysis for overall survival in the study population.

	Univariate		Multivariate			
			All Cohort		HBV, HCV Cohort	
	HR (95% CI)	*p*-Value	HR (95% CI)	*p*-Value	HR (95% CI)	*p*-Value
Whole Model	NA			<0.001		<0.001
Male gender	1.13 (1.109–1.156)	<0.001	1.17 (1.143–1.193)	<0.001	1.19 (1.156–1.217)	<0.001
Age	1.02 (1.019–1.021)	<0.001	1.01 (1.012–1.013)	<0.001	1.00 (1.003–1.005)	<0.001
Cirrhosis	1.07 (1.044–1.089)	<0.001	1.00 (0.980–1.023)	0.891	1.13 (1.102–1.168)	<0.001
Diabetes mellitus	1.20 (1.175–1.216)	<0.001	1.03 (1.009–1.046)	0.004	1.04 (1.014–1.058)	0.001
Hypertension	1.15 (1.128–1.167)	<0.001	0.77 (0.759–0.789)	<0.001	0.77 (0.752–0.787)	<0.001
Dyslipidemia	0.86 (0.841–0.871)	<0.001	0.76 (0.749–0.777)	<0.001	0.77 (0.754–0.787)	<0.001
CCI	1.21 (1.202–1.210)	<0.001	1.120 (1.192–1.200)	<0.001	1.22 (1.213–1.224)	<0.001
HCC treatment, curative vs. non-curative	0.23 (0.223–0.236)	<0.001	0.26 (0.254–0.269)	<0.001	0.26 (0.254–0.271)	<0.001
AFP number	0.93 (0.930–0.936)	<0.001	0.94 (0.940–0.947)	<0.001	0.96 (0.952–0.960)	<0.001
Antiviral treatment	0.71 (0.692–0.724)	<0.001	NA		0.88 (0.860–0.905)	<0.001

Abbreviations: HR, hazard ratio; CI, confidence interval; NA, not available; CCI, Charlson Comorbidity Index; HCC, hepatocellular carcinoma; AFP, alpha-fetoprotein.

## Data Availability

The datasets generated and/or analyzed during the current study are available from the corresponding author upon reasonable request.

## Supplementary Materials

The following supporting information can be downloaded at: https://www.mdpi.com/article/10.3390/cancers16010150/s1, Table S1: List of KDATCC Codes and ICD-10 Codes.
