# Supplementary material for: Regular Alpha-Fetoprotein Tests Boost Curative Treatment and Survival for Hepatocellular Carcinoma Patients in an Endemic Area
Source: Cancers (Basel). 2023 Dec 28;16(1):150. doi: 10.3390/cancers16010150 (PMC10778056; doi:10.3390/cancers16010150)
Supplement: Supplementary file 1 [file cancers-16-00150-s001.zip › cancers-2755994-supplementary.pdf]

Supplementary Table S1. List of KDATCC Codes and ICD-10 Codes

**Name**	**KDATCC codes**
Lamivudine	180901ATB
Adeofovir	457501ATB
Entecavir	487202ATB, 487202ATD, 487203ATB, 487203ATD
Tenofovir disoproxil fumarate	493901ATB, 664901ATB
Tenofovir alafenamide	665301ATB
Tenofovir disoproxil asparate	665101ATB
Telbivudine	506001ATB
Clevudine	487801ACH, 487802ACH, 487803ACH
Besifovir	665401ATB
Ribavirin	223604ACH, 223601ACH, 223601ACH
Interferon	L03AB04, L03AB05, L03AB10, L03AB11
**Name**	**ICD-10 codes**
HBV	Z22.51, B18.0, B18.1, B18.10, B18.18
HCV	B18.2, Z22.52
Alcohol	K70.0, K70.1, K70.2, K70.3, K70.4, K70.9
Non-alcoholic fatty liver disease	K76.0, K76.0A, K75.8B
Others	
Wilson's disease	E83.0
Autoimmune hepatitis	K75.4
Primary biliary cholangitis	K74.30, K74.31, K74.32, K74.39
Liver cirrhosis	K74.0X, K74.1X, K74.2X, K74.3X, K74.4X, K74.5X, K74.6X, K70.2, K70.3, K76.6, I85.XX, I86.4X, K70.43, K71.11, K72.01, K72.11, K72.91
